# Screening for Significant Refractive Error Using a Combination of Distance Visual Acuity and Near Visual Acuity

**DOI:** 10.1371/journal.pone.0117399

**Published:** 2015-02-17

**Authors:** Peiyao Jin, Jianfeng Zhu, Haidong Zou, Lina Lu, Huijuan Zhao, Qiangqiang Li, Xiangui He

**Affiliations:** 1 Department of Preventative Ophthalmology, Shanghai Eye Disease Prevention and Treatment Center, Shanghai, China; 2 Department of Ophthalmology, Shanghai First People’s Hospital, Shanghai Jiao Tong University, Shanghai, China; 3 Department of Eye Disease Prevention and Control, Baoshan Center for Disease Prevention and Control, Shanghai, China; Bascom Palmer Eye Institute, University of Miami School of Medicine;, UNITED STATES

## Abstract

**Purpose:**

To explore the effectiveness of using a series of tests combining near visual acuity (NVA) and distance visual acuity (DVA) for large-scale screenings for significant refractive error (SRE) in primary school children.

**Method:**

Each participant underwent DVA, NVA and cycloplegic autorefraction measurements. SREs, including high myopia, high hyperopia and high astigmatism were analyzed. Cycloplegic refraction results were considered to be the gold standard for the comparison of different screening measurements. Receiver-operating characteristic (ROC) curves were constructed to compare the area under the curve (AUC) and the Youden index among DVA, NVA and the series combined tests of DVA and NVA. The efficacies (including sensitivity, specificity, positive predictive value, and negative predictive value) of each test were evaluated. Only the right eye data of each participant were analysed for statistical purpose.

**Result:**

A total of 4416 children aged 6 to 12 years completed the study, among which 486 students had right eye SRE (SRE prevalence rate = 11.01%). There was no difference in the prevalence of high hyperopia and high astigmatism among different age groups. However, the prevalence of high myopia significantly increased with the age (χ² = 381.81, p<0.01). High hyperopia was the biggest SRE factor associated with amblyopia（*p*＜0.01，OR = 167.40, 95% CI: 75.14∼372.94). The DVA test was better than the NVA test for detecting high myopia (Z = 2.71, p<0.01), but the NVA test was better for detecting high hyperopia (Z = 2.35, p = 0.02) and high astigmatism (Z = 4.45, p<0.01). The series combined DVA and NVA test had the biggest AUC and the highest Youden Index for detecting high hyperopia, myopia, astigmatism, as well as all of the SREs (all p<0.01).

**Conclusion:**

The series combined DVA and NVA test was more accurate for detecting SREs than either of the two tests alone. This new method could be applied to large-scale SRE screening of children, aged 6 to 12, in areas that are less developed.

## Introduction

Uncorrected significant refractive error (SRE) is a universal and serious problem, particularly in children. According to a recently published study, 7% of preschool children in Taiwan have uncorrected SREs[1]. This not only leads to eyestrain, headaches and poor academic performance, but can also result in amblyopia[2]. Although screening programs for refractive errors are carried out all over China, the outcomes of these screenings are affected by several factors. Firstly, refractive error that is successfully detected by the screening programs is not always taken seriously enough. Some parents and children neglect the advice of health care workers because they believe that low grade refractive error is very common and harmless. However, if SREs are detected, particularly in children, specific warnings regarding amblyopia risks may help to increase awareness. Secondly, in some rural areas of China, a large number of children under 6 years old do not attend preschool, thus screening programs are not accessible. According to the 2010 national census in China, 39% of the population of Shanghai immigrated from other less developed provinces in China. It has been reported that the risk of finding an undiagnosed, significant eye disorder in a school entrant was more than 6 times greater for a child who was not examined during the preschool years, and the risk of finding an amblyopic child was more than 10 times greater[3]. Therefore, it is vital that primary school students in less developed areas are screened for SREs.

Cyclopledic autorefraction is a widely used method for diagnosing refractive error, however, due to the length of time it takes to induce cyclopledia, it is seldom used for the screening of large populations[4]. Currently, noncycloplegic autorefraction is used for refractive error screening in most primary schools in urban areas of Shanghai[5]. However, in some less developed districts, an uncorrected distance visual acuity test (DVA) is the only screening method used in schools[6]. Previous studies have reported that although the DVA test has a high sensitivity and specificity for detecting myopia, it cannot accurately detect hyperopia and astigmatism. Thus, the DVA test alone is not satisfactory for SRE screening[7,8]. The uncorrected near vision (NVA) test is a simple and useful eye examination with many benefits. It is most commonly used for detecting presbyopia in older people[9]. A recently published study indicated that the NVA test is suitable for the detection of amblyopia risk factors and for low vision examinations in children[10]. Therefore, in the current study, a method combining DVA and NVA was used for the detection of SREs in children.

To the best of our knowledge, there have been few studies that have examined using a combination of NVA and DVA for SRE detection in schoolchildren. The purpose of the present study was to explore the effectiveness of using a series combined test of NVA and DVA for large-scale SRE screening in primary schools.

## Methods

### Setting and participants

The present study is part of a trial, which aims to establish archives of refractive development of children in Shanghai, China. One ophthalmologist, three optometrists, and two public health doctors conducted the study. In total, 7 primary schools from the Baoshan district, a suburban district of Shanghai, were included. Students in grades 1 to 5, aged 6 to 12 years participated in the study. Those students who didn't consented to, or were not suitable for cyclopaedia, were excluded.

This study was conducted according to the tenets of the Declaration of Helsinki and was approved by the institutional review board of the Shanghai Eye Disease Prevention and Treatment Centre. Written informed consent was received from the parents of all children who were enrolled in the study.

### Screening procedure and study outcomes

The screening procedure contained several items. Firstly, DVA and NVA were examined monocularly in each student. A professional ophthalmologist then conducted a detailed ophthalmic examination, including a slit lamp examination and intraocular pressure (IOP) measurement. Lastly, if a student consented to, and was suitable for cyclopledia (i.e. no allergy to tropicamide, anterior chamber angle was not narrow and IOP was not high), he/she underwent cyclopledic autorefraction conducted by an optometrist.

Trained vision-screening technicians examined DVA and NVA, using the Standard Logarithmic Distance Visual Acuity E Chart (Weheng wh01, GuangZhou WeiShiKang) and the Standard Logarithmic Near Visual Acuity E Chart (Wehen 8620-37630978), which are the most commonly used visual acuity charts in China. This procedure has been thoroughly described in a previous paper[5]. DVA and NVA were performed monocularly, without optical aids, at 5 m and 40 cm respectively. The right eye was always examined first. Students were required to identify each optotype within 5 seconds and they were not allowed to squeeze their eyes or squint during the examination.

One drop of 0.5% tropicamide (Wuxi Shanhe Group, Wuxi, China) was instilled to both eyes, every 5 minutes (5 drops total), to induce cyclopledia. Tropicamide is the most commonly used cycloplegic agent in China and many researchers have indicated that it works well, particularly in subjects with dark irises[11,12]. Autorefraction measurements were obtained using a table-mounted KR-8800 (Topcon, Tokyo, Japan) after adequate cyclopledia was achieved. Adequate cyclopledia was defined as pupil dilation > 6 mm and an absent light reflex. Spherical equivalent refraction (sphere+1/2 cylinder) was used to classify refraction status.

High myopia, high hyperopia, and high astigmatism, were analyzed in this study and were defined as spherical equivalent degree ≤−3.0 dioptres (D), spherical equivalent degree ≥ +4.5 D, and cylinder degree ≥ 2.0 D, respectively[1,4]. Cyclopledic refraction results were considered to be the gold standard when comparing among different screening measurements. The definition of amblyopia was taken from the Refractive Error Study in Children (RESC) surveys. Amblyopia was considered to be a best-corrected visual acuity ≤ 20/40, with no apparent organic lesion, if one or more of the following criteria were met: (1) esotropia, exotropia, or vertical tropia at 4-m fixation or exotropia or vertical tropia at 0.5 m, (2) anisometropia of 2.00 SED or more, or (3) bilateral ametropia of at least +6.00 spherical equivalent degrees.

### Statistical analysis

Two staff members independently input all data into an Epidata 3.1 database. The general characteristics of all participants were analyzed using SAS (version 9.3, SAS Institute, Cary, NC, USA). Categorical variables were analysed by the Chi-square test. Cochran-Armitage Trend Test was used to determine the trend among age groups. Stepwise multiple logistic regression analysis was used to determine whether SREs were associated with amblyopia (inclusion p value = 0.05 and exclusion p value = 0.05). Since DVA, NVA, and cyclopledic autorefraction results were closely related between the right and the left eyes (Spearman correlation = 0.81, 0.71, and 0.91 for DVA, NVA and cyclopledic autorefraction respectively, p<0.001), the right eye was chosen for data analysis. Receiver-operating characteristic (ROC) curves were constructed to compare the area under the curve (AUC) and the Youden index between DVA, NVA and the series combined tests of DVA and NVA by MedCalc (version 13). The best screening criterion was defined as the point closest to the top left-hand corner of the ROC curve[13]. The efficacies (including sensitivity, specificity, positive predictive value and negative predictive value) of each test were evaluated. Results were presented as the mean ± standard deviation for continuous variables and as rates (proportions) for the categorical data. All reported p values are two-sided. p<0.05 was considered statistically significant.

## Results

A total of 5214 participants were recruited for this study, among which 4416 completed all examinations and were included in the analysis. Children were excluded if they were unwilling to accept cyclopledia (n = 778), were unsuitable for cyclopaedia (n = 18) or were uncooperative during the exams (n = 2).

### Prevalence of SREs in different age groups

The mean age of included participants was 8.21±1.73 years old and 54.53% were male (n = 2408). Overall, 486 students with right eye SRE were diagnosed by cyclopledic autorefraction (SRE prevalence rate = 11.01%), including 6 with high hyperopia and astigmatism, 30 with high myopia and astigmatism, 32 with high hyperopia alone, 347 with high myopia alone and 71 with high astigmatism alone. The prevalence of each disease in different age groups were shown in Table 1. Although there were no differences in the prevalence of hyperopia and astigmatism among different age groups, the prevalence of myopia significantly increased with increasing age (χ² = 381.81, p<0.01), and increased fastest in children from 9 years old to 11 years old (partition of Chi-square analysis, p<0.01). Although the prevalence of SREs increased with the age (χ² = 272.21, p<0.01), the prevalence of amblyopia was stable among different age groups (Table 1). Among 60 students with amblyopia, 22 had high myopia (including 2 had high myopia and high astigmatism), 16 had high hyperopia (including 1 had high hyperopia and high astigmatism), and 5 had high astigmatism alone. The remaining 17 students were suffering from other diseases such as strabismus and congenital cataract. Stepwise multiple logistic regression analysis indicated that high hyperopia was the biggest SRE factor associated with amblyopia(*p*<0.01, OR = 167.40, 95%CI: 75.14∼372.94), followed by high astigmatism(*p*<0.01, OR = 17.44, 95%CI: 6.25∼48.68), and high myopia(*p*<0.01, OR = 14.27, 95%CI: 7.51∼27.11). There was no difference in the prevalence of high hyperopia, high myopia, high astigmatism or amblyopia between males and females in any age group (p>0.05).

**Table 1:** The prevalence of significant refractive error in different age groups: number (rate).

age	All	6	7	8	9	10	11	12	p
Number of children	4416	929	815	798	814	601	232	227	
High Hyperopia*	38 (0.86)	13 (1.40)	4 (0.49)	5 (0.63)	7 (0.86)	5 (0.83)	4 (1.72)	0 (0.00)	0.39
High Myopia^§^	377 (8.54)	6 (0.65)	20 (2.45)	48 (6.02)	69 (8.48)	98 (16.31)	67 (28.88)	69 (30.40)	<0.01
High Astigmatism^¥^	71 (1.61)	18 (1.94)	19 (2.33)	7 (0.88)	16 (1.97)	7 (1.16)	1 (0.43)	3 (1.32)	0.08
All SREs	486 (11.01)	37 (3.98)	43 (5.28)	60 (7.52)	92 (11.30)	110 (18.30)	72 (31.03)	72 (31.72)	<0.01
Amblyopia	60 (1.36)	15 (1.61)	10 (1.23)	8 (1.00)	10 (1.23)	8 (1.33)	4 (1.72)	5 (2.20)	0.70

Note: *High hyperopia: spherical equivalent degree ≥ +4.5 dioptres, including 6 students with high hyperopia and astigmatism, and 32 students with high hyperopia alone.

^§^High myopia: spherical equivalent degree ≤−3.0 dioptres, including 30 with high myopia and astigmatism, and 347 with high myopia alone.

^¥^High Astigmatism: cylinder degree ≥ 2.0 D with no significant spherical degree

### Receiver operating characteristic curve analysis

**Fig 1:**
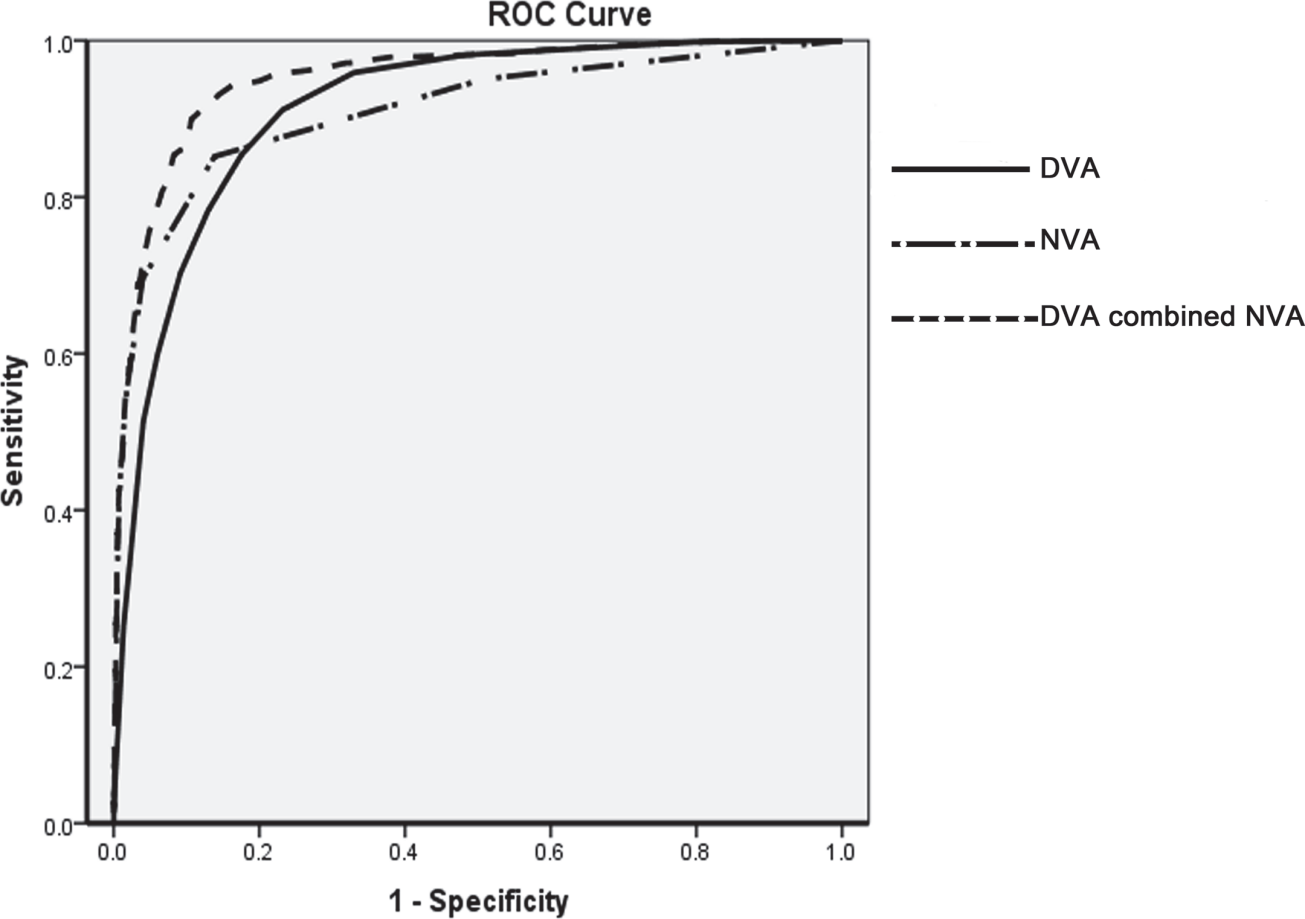
The receiver operating characteristic (ROC) curve of distance visual acuity (DVA), near visual acuity (NVA) and the series combined test of distance and near visual acuity (DVA combined NVA) in detecting significant refractive errors.

The ROC curves and the best cutoff points of DVA, NVA and series combined vision tests are shown in Table 2. The AUC differences among different screening methods were tested, and the significance levels are shown in Table 3. The AUC of the DVA test was larger than that of the NVA test when detecting high myopia in children; however, the NVA test had a much larger AUC than that of the DVA test when detecting high hyperopia and astigmatism (Tables 2 and 3). The series combined test of DVA and NVA had the biggest AUC when detecting high hyperopia, myopia, astigmatism, as well as all SREs (Fig. 1, Tables 2 and 3). The Youden Index, which describes the accuracy of a test, also confirmed that DVA is better than NVA for high myopia screening and that NVA is better than DVA for detecting high hyperopia and astigmatism. Furthermore, the Youden Index of the series combined test was higher than that for the DVA or NVA test alone for detecting high hyperopia, myopia and astigmatism, as well as for detecting all SREs (Tables 2 and 3). The sensitivities and specificities for the detection of each disease by each test are listed in Table 2. The positive predictive value (PPV) and the negative predictive value (NPV) of the series combined test for detecting all SREs were 58.25 and 97.85, respectively. This indicated that, with referral criteria of DVA≤20/32 and NVA≤20/25, almost 60% of referred children, and only 2% of excluded children were actual SRE patients.

**Table 2:** The receiver-operating characteristic (ROC) curve efficacies and cutoff points of near vision acuity (NVA), distance vision acuity (DVA) and the series combined test.

Disease	Method	AUC*	criterion	YI^§^	Se. ^¥^	Sp.^＄^	PPV^※^	NPV^＃^
High hyperopia (N = 38)	DVA	0.77	≤20/32	0.50	89.5	60.6	1.93	99.85
	NVA	0.85	≤20/25	0.60	81.6	78.9	3.24	99.80
	Combined	0.86	DVA≤20/32 NVA ≤20/25	0.64	78.9	84.8	4.30	99.78
High myopia (N = 377)	DVA	0.95	≤20/50	0.79	97.1	81.7	33.15	99.67
	NVA	0.91	≤20/25	0.70	85.9	84.3	33.86	98.47
	Combined	0.97	DVA≤20/63 NVA ≤20/20	0.79	87.8	90.7	46.95	98.76
High astigmatism (N = 107)	DVA	0.77	≤20/32	0.48	86.9	61.3	5.28	99.47
	NVA	0.85	≤20/25	0.63	83.2	79.9	9.30	99.48
	Combined	0.87	DVA≤20/25 NVA ≤20/25	0.64	81.3	83.1	10.65	99.44
All SREs^○^ (N = 486)	DVA	0.91	≤20/40	0.70	91.2	76.8	32.69	98.60
	NVA	0.91	≤20/25	0.71	85.2	86.2	43.26	97.92
	Combined	0.95	DVA≤20/32 NVA ≤20/25	0.76	83.5	92.6	58.25	97.85

Note: *AUC: area under the curve

^§^YI: Youden Index

^¥^Se.: Sensitivity

^＄^Sp.: Specificity

^※^PPV: positive predictive value

^＃^NPV: negative predictive value

^○^SREs: significant refractive errors

**Table 3:** The significance level (P) and Z statistics of the differences between distance vision acuity (DVA), near vision acuity (NVA) and combined examinations: P (Z).

		AUC*	DVA	NVA	Combined
High hyperopia (N = 38)	DVA	0.77	-	0.02 (2.35)	<0.01 (2.79)
	NVA	0.85	0.02 (2.35)	-	0.68 (0.41)
	Combined	0.86	<0.01 (2.79)	0.68 (0.41)	-
High myopia (N = 377)	DVA	0.95	-	<0.01 (2.71)	0.86 (0.17)
	NVA	0.91	<0.01 (2.71)	-	<0.01 (3.08)
	Combined	0.97	0.86 (0.17)	<0.01 (3.08)	-
High astigmatism (N = 107)	DVA	0.77	-	<0.01 (4.45)	<0.01 (4.62)
	NVA	0.85	<0.01 (4.45)	-	0.56 (0.58)
	Combined	0.87	<0.01 (4.62)	0.56 (0.58)	-
All SREs^○^ (N = 486)	DVA	0.91	-	0.58 (0.55)	<0.01 (7.77)
	NVA	0.91	0.58 (0.55)	-	<0.01 (9.23)
	Combined	0.95	<0.01 (7.77)	<0.01 (9.23)	-

Note: *AUC, area under the curve

^○^SREs, significant refractive errors

## Discussion

The results of this study indicate that the prevalence of high myopia significantly increases with age; however, there was no difference in the prevalence of high hyperopia and astigmatism among different age groups. High hyperopia was the biggest SRE factor associated with amblyopia. DVA examination is a commonly used screening method, but has shortfalls in detecting high hyperopia and astigmatism. NVA examination is much better for detecting high hyperopia and astigmatism and could be a good complement to DVA examination. The series combined test of DVA and NVA enables better detection of all SREs than either examination alone.

In total, 11.01% of the children were diagnosed with SREs in the right eye and only the prevalence of high myopia increased with age. This finding is consistent with a previous study conducted in China[14]. In our study, the prevalence of high hyperopia, myopia and astigmatism were 0.86%, 8.54%, and 2.42%, respectively. The prevalence of high myopia among participants in the current study was much higher compared to previous research conducted in preschool children in Taiwan[1], in which the prevalence of high hyperopia, myopia and astigmatism were 1.0%, 0.6%, and 5.1%, respectively. It is likely that the higher prevalence of myopia observed in the current study is due to the older age of participants. The astigmatism prevalence in the current study was lower than that reported in preschool children in Taiwan, though the result is consistent with a recently reported study, in which the prevalence of astigmatism was found to be lower in older children[15,16].

DVA is the most widely used method of refractive error screening in China and worldwide[17]. DVA is highly accurate for detecting significant myopia (AUC = 0.95); however, it is not accurate when screening for high hyperopia and astigmatism (AUC<0.8). The NVA test provides much better accuracy when screening for high hyperopia and astigmatism (AUC = 0.85), but is an ineffective method for detecting myopia. Former epidemiological surveys have reported a high prevalence of both astigmatism and hyperopia in Chinese children[18,19], which are also known risk factors for the development of strabismus and amblyopia[20,21]. Our result indicated that among common SRES, high hyperopia was the most important cause of amblyopia. Thus, combining DVA and NVA in SRE screenings is important and necessary. Moreover, the series combined NVA and DVA test largely increased the specificity without significantly lowering the sensitivity. Since the prevalence of SREs is not low in primary school students, increasing the specificity could largely decrease unnecessary referrals, thus reducing the cost. During the screening, children with a DVA less than 20/32 would have an additional NVA examination, and children with an NVA less than 20/25 would be suspected of having an SRE. These children and their parents would then be notified. Parents who do not realize the importance of uncorrected refractive error in their children would be given information, and warned about the potential for the development of strabismus and amblyopia, which would likely increase compliance with necessary treatments.

Although the series combined test of DVA and NVA was not as good as noncycloplaegic autorefraction and photorefraction for detecting low refractive errors[5,22], its accuracy was satisfactory for SRE screening. Furthermore, noncycloplaegic autorefraction and photorefraction require expensive equipment and experienced operators, while DVA and NVA tests are easier to carry out and are much less expensive. School-teachers can be trained to administer DVA and NVA tests and since the examinations are done quickly and cause no discomfort, students and their parents easily accept them. Unlike noncycloplaegic autorefraction and photorefraction equipment, DVA and NVA equipment is small and easily portable, which is extremely valuable when travelling to regions that lack convenient transportation. These characteristics make the series combined DVA and NVA test appropriate for SRE screening in less developed areas.

There were some limitations to the current study. Firstly, the referral criteria of the series combined test might not effectively apply to other populations. Secondly, with different cycloplegic agents, the diagnosis of SRE might be a little biased in different population. Because although tropicamide was proved to be a useful cycloplegic agent in Asian population, some former studies indicated that the cycloplegic effect of tropicamide was much less than that of cyclopentolate or atropine in Caucasian population[23]. Thirdly, since the study population was not randomly selected, the representativeness of the result could be affected.

In conclusion, the series combined test of DVA and NVA had better accuracy when screening for SREs than either of the tests alone. This new method could be used for large-scale SRE screening of children, aged 6 to 12 years, who reside in less developed areas.
